# TGF-ß1 Induces Changes in the Energy Metabolism of White Adipose Tissue-Derived Human Adult Mesenchymal Stem/Stromal Cells *In Vitro*

**DOI:** 10.3390/metabo10020059

**Published:** 2020-02-07

**Authors:** Olga Hahn, Lena-Christin Ingwersen, Abdelrahman Soliman, Mohamed Hamed, Georg Fuellen, Markus Wolfien, Julia Scheel, Olaf Wolkenhauer, Dirk Koczan, Günter Kamp, Kirsten Peters

**Affiliations:** 1 Department of Cell Biology, Rostock University Medical Center, 18057 Rostock, Germany; olga.hahn@med.uni-rostock.de (O.H.); lena-christin.ingwersen@med.uni-rostock.de (L.-C.I.); soliman_1993@live.com (A.S.); 2 Institute for Biostatistics and Informatics in Medicine and Ageing Research, Rostock University Medical Center, 18057 Rostock, Germany; mohamed.hamed@uni-rostock.de (M.H.); fuellen@uni-rostock.de (G.F.); 3 Department of Systems Biology and Bioinformatics, University of Rostock, 18057 Rostock, Germany; markus.wolfien@uni-rostock.de (M.W.); julia.scheel@uni-rostock.de (J.S.); olaf.wolkenhauer@uni-rostock.de (O.W.); 4 Core Facility for Microarray Analysis, Rostock University Medical Center, 18057 Rostock, Germany; dirk.koczan@med.uni-rostock.de; 5 AMP-Lab GmbH, 48149 Münster, Germany; kamp@amplab.de

**Keywords:** TGF-ß1, adipose tissue-derived mesenchymal stem/stromal cells, energy metabolism, gene expression analyses

## Abstract

Adipose tissue plays an active role in the regulation of the body’s energy balance. Mesenchymal stem/stromal cells from adipose tissue (adMSC) are the precursor cells for repair and adipogenesis. Since the balance of the differentiation state of adipose tissue-resident cells is associated with the development of various diseases, the examination of the regulation of proliferation and differentiation of adMSC might provide new therapeutic targets. Transforming growth factor-β1 (TGF-ß1) is synthetized by many cell types and is involved in various biological processes. Here, we investigated the effects of different concentrations of TGF-ß1 (1–10 ng/mL) on adMSC proliferation, metabolic activity, and analyzed the gene expression data obtained from DNA microarrays by bioinformatics. TGF-ß1 induced the concentration- and time-dependent increase in the cell number of adMSC with simultaneously unchanged cell cycle distributions. The basal oxygen consumption rates did not change significantly after TGF-ß1 exposure. However, glycolytic activity was significantly increased. The gene expression analysis identified 3275 differentially expressed genes upon exposure to TGF-ß1. According to the pathway enrichment analyses, they also included genes associated with energy metabolism. Thus, it was shown that TGF-ß1 induces changes in the energy metabolism of adMSC. Whether these effects are of relevance in vivo and whether they contribute to pathogenesis should be addressed in further examinations.

## 1. Introduction

White adipose tissue plays an active role in the regulation of the energy balance of the body by storing and mobilizing lipids and by affecting the glucose level [1,2]. Furthermore, cells of the adipose tissue are responsible for the release of a vast number of so-called adipokines such as leptin or adiponectin, which are involved in the regulation of lipid metabolism, but also in the pathogenesis of diseases such as type 2 diabetes or arteriosclerosis [3]. Besides the characteristic adipocytes, adipose tissue contains other cell types such as preadipocytes, macrophages, lymphocytes, endothelial cells, and, as in most other connective tissue, also mesenchymal stem/stromal cells (MSC) [4,5,6]. Adipose tissue-derived MSC (adMSC) are the tissue-specific precursor cells for repair and adipogenesis and were first described by Zuk et al. [7]. In general, the MSC contribute to tissue homeostasis and regeneration, since they have an intrinsic capacity for self-renewal and for differentiation into various mesenchymal cell lineages including chondrocytes, osteocytes, adipocytes, cardiomyocytes, and smooth muscle cells [8,9]. MSC are of therapeutic interest due to the broad range of differentiation described and their immunomodulatory potential [4,8]. The balance between the different adipose tissue-resident cell types and their differentiation state is closely related to the maintenance of energy homeostasis. Increases in the size and number of adipocytes are associated with the development of various diseases, such as metabolic syndrome [10]. Thus, the examination of the regulation of proliferation and differentiation of preadipocytes and adMSC, and understanding of the interrelationship between the different cell types will provide new targets for action against these diseases [11].

The differentiation of MSC is controlled by specific growth and transcription factors; among them, the members of the transforming growth factor-β (TGF-β) family [8]. The TGF-β family members include TGF-β1, β2, and β3, bone morphogenetic proteins (BMPs), and various others. The family is named after its first member, the TGF-β1, originally described in 1983 [12]. It is the prevalent protein isoform, which is synthetized by many cell types, e.g., macrophages, keratinocytes, and chondrocytes [13]. The TGF-ß family members are involved in many biological processes due to their pleiotropic effects. These include ontogenesis and cell differentiation, wound healing, hematopoiesis, and immune response [14]. Various studies have shown that TGF-ß1 induces contradictory effects, e.g., by either increasing or reducing proliferation depending on the MSC sources and the TGF-ß1 concentration applied [15,16,17]. Furthermore, the effects of TGF- β1 are dependent on the environment (tissue and cell neighborhood) and the exposure time [17]. TGF-ß1 plays a significant role in myofibroblastic differentiation. This cell type plays an essential role in wound healing, pathological organ remodeling, general mechanisms of extracellular matrix synthesis, and tension production [18]. Myofibroblasts can evolve from mesenchymal cells through transdifferentiation caused by three local events: the presence of a specialized extracellular matrix, high mechanical stress, and the accumulation of TGF-ß1 [19,20,21,22]. 

The energy metabolism of stem cells is adapted to meet the demands for their diverging functions and needs [23,24]. The differentiation of MSC can be accompanied by changes in their energy metabolic characteristics. Thus, it could be shown that adMSC in adipogenic differentiation undergo a shift of energy metabolism towards oxidative phosphorylation [25]. Furthermore, the osteogenic differentiation of bone marrow MSC was affected by inhibitors of the mitochondrial oxidative phosphorylation without a reduction of ATP levels. Thus, it is suggested that active mitochondria support osteogenic differentiation not only by supplying ATP [26]. However, the number of studies regarding the energy metabolic characteristics of MSC in differentiation is sparse, especially when considering high throughput gene expression datasets.

Since TGF-ß1 holds a pivotal role in tissue regeneration and adMSC are known to respond after TGF-ß1 exposure, we examined the effects of TGF-ß1 on adMSC in vitro regarding cell proliferation and cell cycle, metabolic activity, including mitochondrial activity and acidification, and gene expression.

## 2. Results

### 2.1. Quantification of the Cell Number and Metabolic Activity

**Figure 1:**
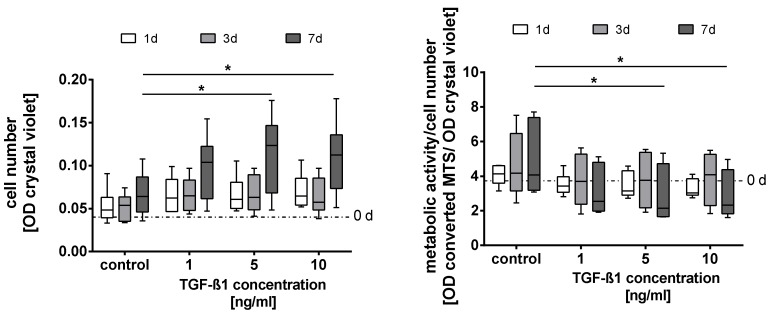
Cell number (**a**) and metabolic activity per cell number (**b**) after exposure to different concentrations of TGF-ß1 on day 0, 1, 3, and 7 (cell number quantified with crystal violet staining, metabolic activity measured via a tetrazolium salt conversion assay). Data presented as box plots with medians, interquartile ranges and minimum/maximum values (*n* = 6). Since the dataset did not represent a Gaussian distribution (Shapiro-Wilk test), the statistical analysis was performed using the Two-Way variance analysis test ANOVA followed by Dunnett’s multiple comparison post hoc test. * *p* ≤ 0.05. Comparison with the control.

The treatment of adMSC with TGF-ß1 did not induce significant changes in the cell number on day 1 and 3 compared with the untreated controls (Figure 1a). After 7 days of exposure, the higher TGF-ß1 concentrations (5 ng/mL and 10 ng/mL) induced a significant increase in the cell number, as compared with the control. No significant difference was found between the values for 5 ng/mL and 10 ng/mL TGF-ß1. In accordance with the cell number, the relative metabolic activity of day 1 and 3 remained similar to the untreated control (dataset in Appendix A). After 7 days of cultivation, all TGF-ß1 concentrations induced a decrease of the metabolic activity per cell number compared with the control cultures, whereby only measurements for higher TGF-ß1 concentrations (5 ng/mL and 10 ng/mL) reached statistical significance (Figure 1b).

### 2.2. Cell Cycle Analyses

The analyses of the cell cycle after TGF-ß1 exposure were executed on days 0, 1, 3, and 7 with 10 ng/mL TGF-ß1. The results of all days are depicted in Table 1. The TGF-ß1 exposure exhibited no significant differences in the sub G1, G0/G1, S, and G2 phases of the cell cycle analysis. The control cultures as well as the TGF-ß1 cultures revealed similar values for each cell cycle phase. This can be observed for all measured time points. Thus, the increase in cell numbers shown above are not associated with an increase in the cell numbers in a specific cell cycle phase.

### 2.3. Metabolic Characterization 

#### 2.3.1. Quantification of Mitochondrial Respiration and Glycolysis

**Figure 2:**
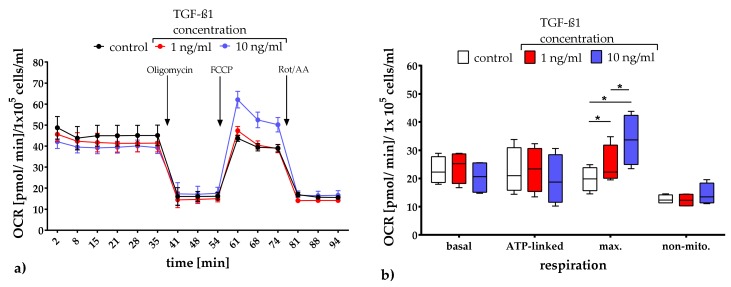
Quantification of the oxygen consumption rate (OCR) after TGF-ß1 exposure on day 3. OCR data presented as mean with the standard error of the mean normalized to 1 × 10^5^ cells/ml (**a**). Corresponding to their function in the respiratory chain, the numerical data were presented as box plots, with medians, interquartile ranges and minimum/maximum values (**b**). Since the dataset did not represent a Gaussian distribution (Shapiro-Wilk test), the statistical analysis was performed using the Two-Way ANOVA test followed by Tukey’s multiple comparison post hoc test (*n* = 4). * *p* ≤ 0.05. Comparison with the control. FCCP: carbonyl-cyanide-4 (trifluoromethoxy) phenylhydrazone; Rot/AA: rotenone/antimycin A; ATP: adenosine triphosphate; max.: maximal; non-mito.: non-mitochondrial.

In order to analyze mitochondrial respiration and glycolysis, a test system for assessing mitochondrial function, basal respiration, ATP production-coupled respiration, non-mitochondrial respiration, and extracellular acidification (Cell Mito Stress Test) was performed on days 1, 3, and 7 (Figure 2). Both culture conditions, i.e., unstimulated controls and TGF-ß1-exposed cultures, revealed a non-significantly but TGF-ß1-concentration dependently decrease of the basal respiration about appr. 12% upon TGF-ß1-exposure (Figure 2a). Furthermore, the inhibition of complex V of the respiratory chain by oligomycin exposure led to a similar decrease of the oxygen consumption rate (OCR) in all culture conditions examined. The exposure of the uncoupling agent FCCP generated an increase in the maximal OCR by complex IV, whereby concentration-dependent differences were apparent. The highest TGF-ß1 concentration [10 ng/mL] induced the highest maximal OCR (mean: 31.94 ± 6.08). In addition, in all cultures the exposure to the combination of rotenone and antimycin A (inhibitors of complex I and III of the respiratory chain) triggered a decrease in mitochondrial respiration; this allows conclusions to be drawn about the non-mitochondrial respiration. In the box plot depiction of OCR according to function in the respiratory chain it is shown that the TGF-ß1 exposure has no influence on the basal, ATP-linked and non-mitochondrial respiration (Figure 2b). However, the maximal respiration was significantly increased in a concentration-dependent manner by adding TGF-ß1 (control vs. 1 ng/mL: *p*-value 0.0124; control vs. 10 ng/mL: *p*-value < 0.0001 and 1 ng/mL vs. 10 ng/ mL: *p*-value < 0.0001). Similar effects could also be shown on day 1 and 7 (dataset in Appendix B).

**Figure 3:**
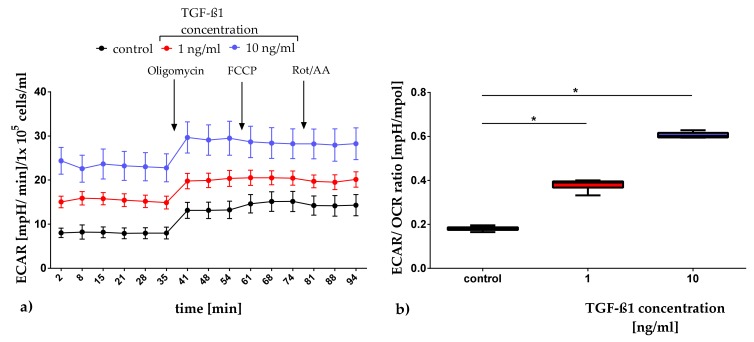
Quantification of the extracellular acidification rate (ECAR) after TGF-ß1 exposure on day 3. ECAR data are presented as a mean, with the standard error of the mean normalized to 1 × 10^5^ cells/ml (**a**). The basal metabolism of the cultures was assessed by calculating ECAR/OCR ratios. Data were presented as box plots, with medians, interquartile ranges and minimum/maximum values (**b**). Since the dataset showed no Gaussian distribution (Shapiro-Wilk test), the statistical analysis was performed using the One-Way ANOVA test followed by Dunnett’s multiple comparison post hoc test (*n* = 4). * *p* ≤ 0.05. Comparison to the control. FCCP: carbonyl-cynaide-4 (trifluoromethoxy) phenylhydrazone; Rot/AA: rotenone/ antimycin A.

Glycolytic activity was analyzed by measuring extracellular acidification, which is presented in Figure 3 as the extracellular acidification rate (ECAR). During basal respiration, the ECAR increases concentration-dependently (Figure 3a). To analyze the basal metabolism of the cell cultures, the ECAR/OCR ratios were calculated. The box plot depiction of this ratio is presented in Figure 3b. Comparing the control cultures with the cultures exposed to TGF-ß1, a significant concentration-dependent increase of the ECAR/OCR ratio was apparent (1 ng/mL: *p*-value < 0.0001 and 10 ng/mL: *p*-value < 0.0001). Similar effects could also be shown on days 1 and 7 (dataset in Appendix C).

#### 2.3.2. Gene Expression Analyses of the Energy and Amino Acid Metabolism

**Figure 4:**
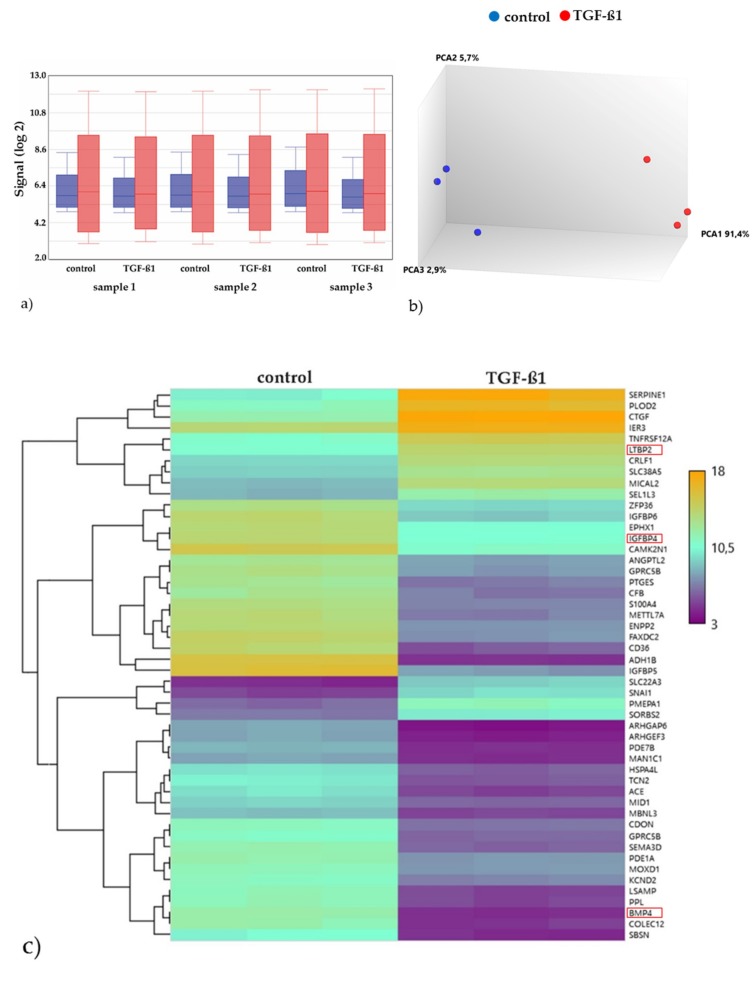
Microarray gene expression analysis after 3 days of cultivation (*n* = 3). Comparison before (blue) and after (red) normalization (**a**). The Principal Component Analysis (PCA) of the controls (blue) vs. TGF-β1 cultures (**b**). Heatmap of the expression patterns of the top 50 differentially regulated genes between control and TGF-ß1 cultures. Violet spots represent lower gene expression, whereas yellow spots denote higher expression. The dendrogram on the left sides shows the hierarchical clustering tree of the genes, respectively (**c**).

The gene expression profiling was performed by a DNA microarray, this allows the expression measure of a large number of genes simultaneously. For this purpose, the fluorescence signal of the phycoerythrin of the entire chip was read by a laser scanner. The signal intensity before (blue) and after normalization (red) demonstrated appropriate data quality (Figure 4a). The Principal Component Analysis (PCA) of the normalized microarray signal intensities revealed distinct groups for the control (blue) and the TGF-β1-exposed cultures (red), which means that the gene expression values of both groups are coherent and are thus suitable for the downstream bioinformatics analysis (Figure 4b). The differential gene expression analysis identifies 3275 significantly differentially expressed genes (1441 up regulated and 1834 down regulated). To show the largest difference between the two sample groups, we visualized the relative expression profiles of the top 50 genes (according to the linear model for microarray data/LIMMA, *p*-value) as a heatmap (Figure 4c). For this purpose, genes with high relative expression values (upregulated) were colored in yellow and genes with low relative expression values (downregulated) were colored in violet, whereas values in between are plotted according to a color gradient. The heatmap provides a visual qualitative representation of the transcriptomic landscape. The expression profiles of the top 50 genes show distinct patterns in both conditions. Examples include the insulin-like growth factor binding protein 5 (IGFBP5), which is involved in cell growth regulation and glucose homeostasis, and the bone morphogenetic protein 4 (BMP4), which is a part of the TGF-ß superfamily and is involved in many biological processes, e.g., bone and cartilage development, adipogenesis, and neurogenesis. Both genes showed lower expression values in the TGF-ß1 cultures compared with the control cultures. On the other hand, the latent transforming growth factor beta binding protein 2 (LTBP2) showed higher expression values compared with the control. The protein encoded by this gene belongs, among others, to the extracellular matrix proteins and is a binding protein for TGF-ß1. 

**Figure 5:**
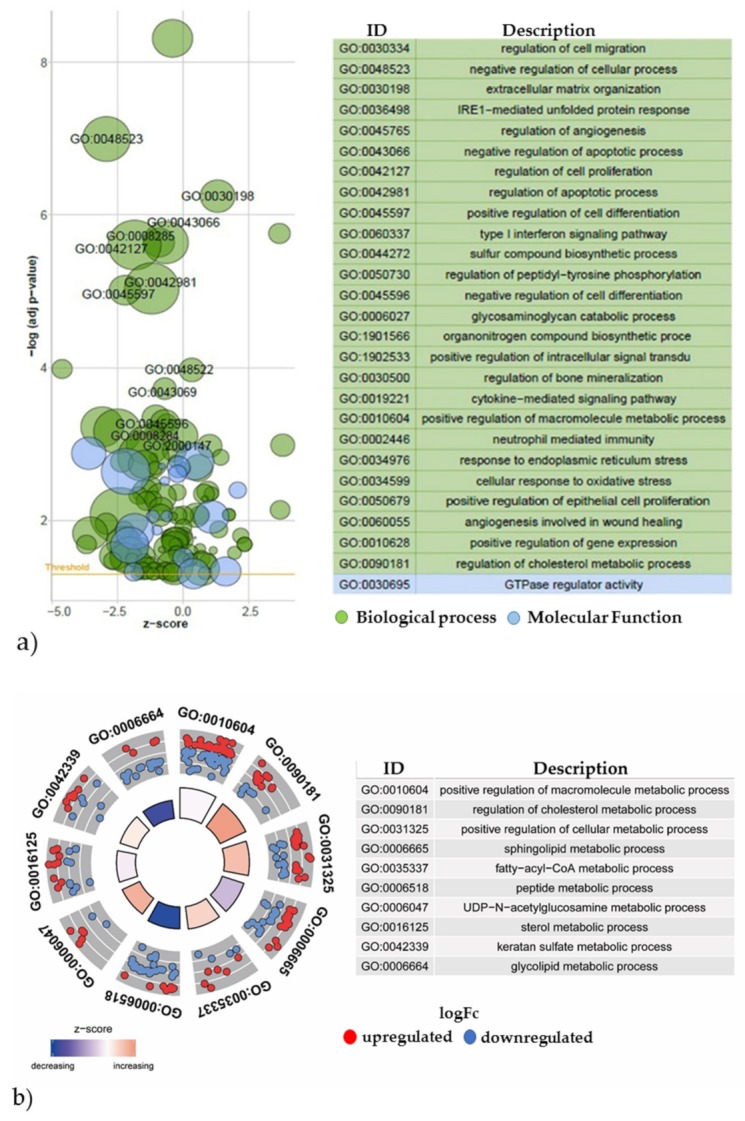

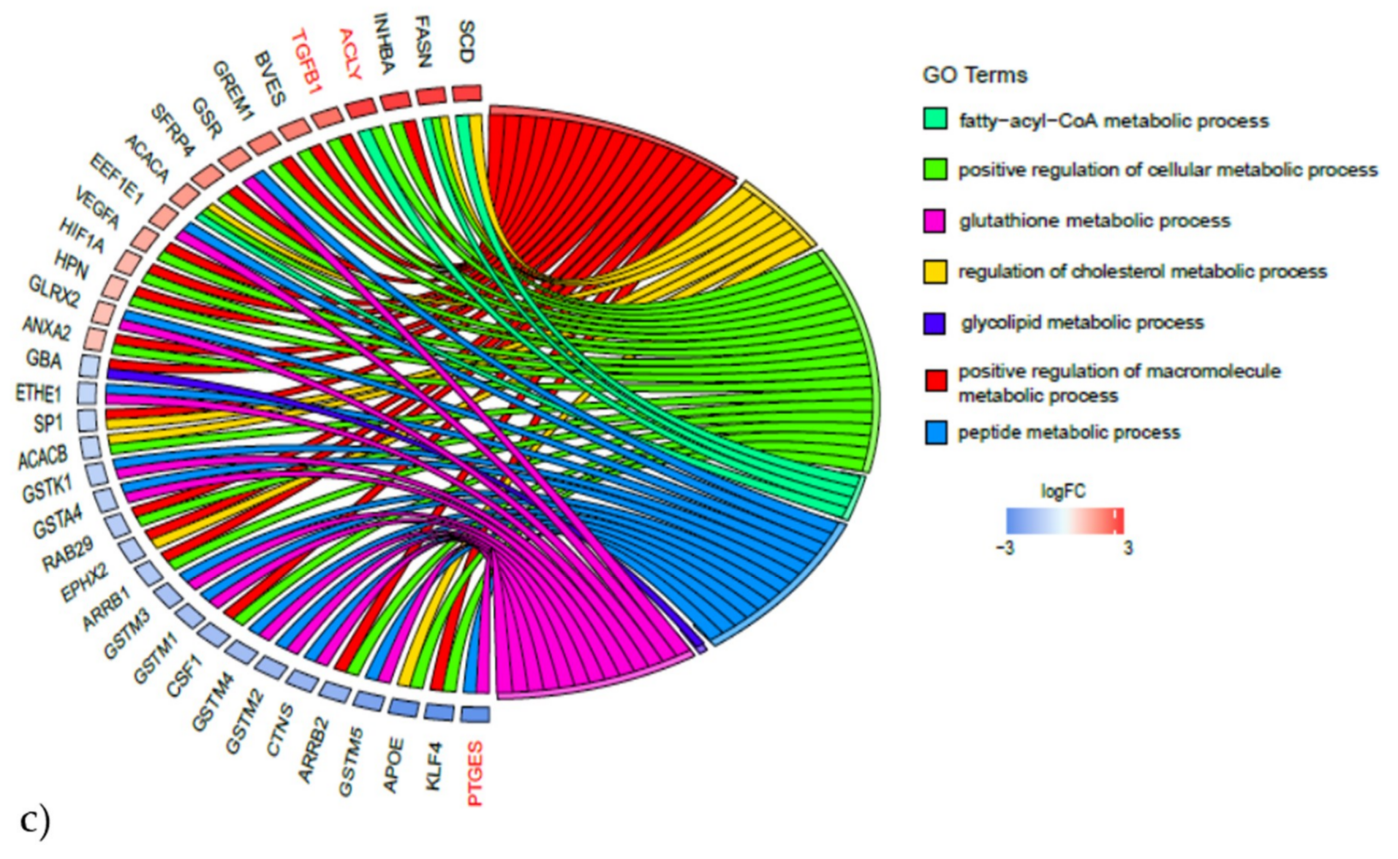
GOplot visualization of significantly regulated, metabolism-related GO terms and the corresponding differentially expressed genes. A selection of the most significantly overrepresented GO terms is shown in a Bubble plot, whereby the z-score is assigned to the x-axis and negative log *P* value to the y-axis. The z-score is indicated by color intensity and the top GO IDs of the respective table are depicted in the plot (**a**). In the GO circle plot the inner ring represents a bar plot, where the bar height indicates the negative log *P* value of the GO term described. The color indicates the z-score and the outer ring shows scatterplots of the log fold change (logFc) of expression levels for genes within the GO term (**b**). Relationship between genes linked to preselected GO terms (**c**). The left section displays the differentially expressed genes, which are associated with the specific metabolic processes, mapped to the right section. The color transition from blue to red indicates the log fold change of the expression levels of the control vs. TGF-ß1 exposed cultures.

This differential analysis enabled us to use the common subsequent approach to deriving insights from a gene expression dataset, which is referred to as gene set enrichment analysis (GSEA) [27]. In this process, differentially expressed genes from genomic, transcriptomic, and proteomics studies are associated with biological processes or molecular functions. For a first overview of the enriched terms, the differentially expressed genes related to metabolism, were plotted as a Bubble Plot (Figure 5a). The x-axis represents the z-score and the y-axis the logarithm of the adjusted *p*-value, whereby each bubble represents a Gene Ontology (GO) term. GO terms include the biological functions at the molecular, cellular and tissue system level of associated specific genes. The number of genes assigned to the GO term and their association with the biological process (green) or molecular function (blue) is proportional to the area and color of the circles, respectively. Here, the biological process related GO terms show a larger distribution of *p*-values compared with the molecular functions GO terms.

Due to the large number of overrepresented GO terms between the control and TGF-ß1 cultures, we preselected terms related to metabolism (as shown in Figure 5a) to obtain an in-depth comparison (Figure 5b). Here, GO circle combines a scatter plot with a bar plot and allows the expression values of the genes assigned to the GO terms to be displayed. The bar height of the inner circle indicates the negative log *p*-value. The color indicates the z-score and the outer circle shows scatter plots of the log fold changes of gene expression levels within each GO term (upregulated: red and downregulated: blue). For example, in the gene expression of genes annotated to GO:0031325, the positive regulation of cellular metabolic processes significantly increases compared with the control cultures. 

To obtain an overview of the relationship between genes linked to multiple processes, we used a GO chord plot. With this representation, differentially expressed genes annotated to specific GO terms could be identified, e.g., the gene ATP citrate lyase (ACLY), significantly upregulated, is involved in fatty-acyl- CoA metabolic processes as well as in the positive regulation of cellular metabolic processes (Figure 5c). Transforming growth factor beta 1 (TGBP1), also significantly upregulated, is associated with a positive regulation of the cellular metabolic process and a positive regulation of the macromolecule metabolic process, whereas prostaglandine E synthase (PTGES), significantly downregulated, is related to the glutathione metabolic process and the peptide metabolic process and is also involved in inflammatory responses.

**Figure 6:**
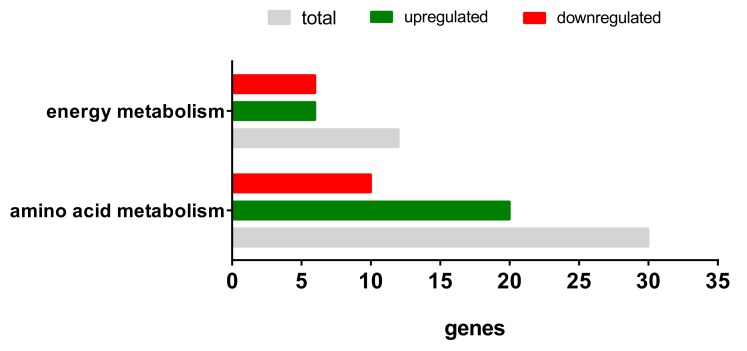
Differentially expressed genes of the energy metabolism and amino acid metabolism after TGF-ß1 exposure.

Based on these results, we took a closer look at two specific pathways (using the Wiki Pathways resource): energy metabolism and amino acid metabolism. An overview of the significantly differentially expressed genes in both pathways is depicted in Figure 6 and listed in Table 2. The TGF-ß1 exposure led to the differential expression of 12 genes involved in the energy metabolism (six upregulated, six downregulated) and 30 regulated genes in the amino acid metabolism (20 upregulated and 10 downregulated. 

## 3. Discussion

Adipose tissue is important in the regulation of the energy balance of the body and therewith involved in the pathogenesis of a number of diseases [1]. Furthermore, the MSC from adipose tissue possess the capacity for self-renewal and multipotent differentiation. Detailed knowledge about the TGF-ß1-induced signaling in adMSC is of high relevance, since TGF-ß1 is involved in many processes of tissue regeneration [14]. Therefore, we determined in the present study the impact of the exposure of recombinant TGF-ß1 on human adMSC in vitro. 

A relationship between TGF-ß1 exposure and the proliferation behavior of MSC has been reported in previous investigations. However, the results were conflicting since some describe an increase in proliferation [28,29,30], whereas others report cell-cycle arrest or cellular senescence [31,32]. The contradictory effects of TGF-ß1 on MSC proliferation appear to be dependent on several different parameters such as tissue and cell environment, the incubation and observation time as well as the applied dose and, even more critical, on the specific cell type and on cell differentiation status [15,16,17,33]. Our results are consistent with Kassem et al. who reported that TGF-ß1 (0.01–10 ng/mL) increased both the DNA synthesis rate as well as the cell number in human bone marrow stromal cells [34]. However, Roostaeian et al. showed a decreased cell proliferation of rabbit bone marrow MSC [35] at a concentration of 10 ng/mL TGF-ß1. 

Furthermore, in our study, no differences regarding the cell cycle phases could be shown, in contrast to earlier findings, where cell cycle arrest or cellular senescence could be detected after TGF-ß1 exposure [31,32]. Approximately 90% of the control as well as the TGF-ß1 cell cultures were in the G0/G1 phase. These findings could be observed for all measured time points. The disagreements with earlier findings may be associated with the different cell sources regarding species (e.g., human vs. rabbit) and tissue (e.g., bone marrow vs. adipose tissue).

In order to characterize energy metabolistic features, we first analyzed the metabolic activity using the a tetrazolium salt conversion assay. Interestingly, all TGF-ß1 concentrations induced a decrease in MTS conversion, thus, indicating reduced mitochondrial activity compared with the control cultures.

Secondly, we examined mitochondrial respiration after TGF-ß1 exposure. The basal respiration decreased dependent on the TGF-ß1-concentration, albeit non-significantly, which indicates a slightly decreased mitochondrial respiration upon TGF-ß1-exposure. This is in agreement with the results from the MTS conversion assay. Conversely, the uncoupling of the respiratory chain revealed a significant and concentration-dependent increase of the maximal respiration capacity upon TGF-ß1 exposure. The TGF-ß1-induced increase of the maximal respiration within the simultaneously slightly reduced basal oxygen consumption is conspicuous. However, Morton et al. also demonstrated a significantly higher maximal respiration capacity of lymphocytes from healthy individuals compared with lymphocytes from acute pancreatitis patients with a simultaneously unchanged basal oxygen consumption [36]. We suggest TGF-ß1 activates a program that increases the maximal respiratory potential of mitochondria, possibly due to mitochondrial mass or architecture, improved fission and fusion, and/or altered reactive oxygen species concentrations [37].

Furthermore, the extracellular acidification rate was significantly and concentration-dependently increased in TGF-ß1-treated adMSC cultures, indicating a higher rate of glycolysis compared with the untreated controls. Since the energy metabolism of adMSC in vitro and in vivo has been shown to be mainly based on glycolysis, the extracellular acidification is most likely by lactate synthesis [38,39]. Earlier studies demonstrated a high glucose consumption accompanied by an increased lactate rate in proliferating bone marrow MSC and adipose-tissue derived MSC [40]. This finding might also correspond to the increased glycolytic preference observed during osteogenic differentiation of adMSC, which is accompanied by increased cell proliferation [25]. Additionally, the pentose phosphate pathway (PPP) has been shown to be of importance in adMSC. The PPP delivers pentoses for RNA or DNA synthesis [40]. Thus, a high PPP capacity has been associated with increased proliferation of various cell lines in vitro [41]. A preference for carbohydrate metabolism to lactate (glycolysis) under aerobic conditions is defined as the Warburg effect [42]. This less efficient energy pathway might be a way to increase the carbon flux through biosynthetic pathways [38,43,44,45].

Interestingly, the expression of the genes ADH1C, ALDH1A1, PPM1L, and PDK4, which are considered to be strongly associated with glycolysis according to the computational differential expression analysis, was significantly downregulated on day 3 after TGF-ß1 exposure. This bioinformatic result, which at first glance appears to be contradictory to the biochemical results regarding an increased glycolytic activity, can be discussed under various aspects: the gene products of the above mentioned genes, such as the alcohol dehydrogenase 1C (ADH1C) and the aldehyde dehydrogenase 1 family member A1 (ALDH1A1), are indirectly associated to glycolysis. Both gene products are involved in alcohol metabolism and the ALDH1A1 is also involved in the regulation of the metabolic response to a high-fat diet [46,47]. In a regulatory context, the protein phosphatase, Mg2+/Mn2+ dependent 1L (PPM1L) is also of interest. The PPM1L protein is located in the endoplasmic reticulum membrane and is involved in adiposity [48,49]. The pyruvate dehydrogenase kinase 4 (PDK4), is another example of a glucose metabolism associated, regulating gene product. The PDK4 gene product links glycolysis to the citric acid cycle and thereby contributes to the regulation of glucose metabolism [50,51]. Therefore, a closer look likewise clarifies these contradictions and highlights the complex regulatory relationships, which was further supported through the findings of the gene set enrichment analysis (GSEA).

The transcriptome and proteome of a cell type rapidly changes in a tightly regulated manner in response to different environmental conditions. Additionally, post-transcriptional and post-translational regulations of gene expression lead to several isoforms or proteoforms with distinct structural and functional attributes originating from the same gene [52]. Thus, the elucidation of the gene expression patterns of adMSC associated with TGF-ß1 on a bioinformatics basis is fundamental to understanding cellular processes and adipose tissue-related diseases. However, the 3275 significantly differentially expressed genes identified give only a first indication of the manifold effects of TGF-ß1 on adMSC. To be able to assess the results presented here, both the energy metabolism and microarray results must be part of further investigations, e.g., on the level of an in-depth metabolite analysis as well as on the protein expression level.

In summary, our study provided further insights into the signaling in adMSC after TGF-β1 treatment, pointing to changes in the energy metabolism and amino acid metabolism. This includes the significant increase of the cell number and the glycolytic activity. Additionally, the bioinformatics analyses provide characterizations of the signaling induced by TGF-ß1 in adMSC. Nevertheless, whether these effects are of relevance in vivo and whether they contribute to pathogenesis should be addressed in further examinations. 

## 4. Materials and Methods 

### 4.1. Cell Isolation and Cultivation

Primary human adipose tissue-derived mesenchymal stem/stromal cells (adMSC) were obtained from seventeen patients (15 female and two male donors with a mean age of 42 ± 11 years and a body mass index of 22.51 ± 3.15 kg/m^2^) undergoing tumescence-based liposuction. Isolation of these cells followed the instructions previously described [53]. Briefly described, the enzymatic digestion with 1.5 U/ml collagenase (NB4 from clostridium histolyticum; Nordmark Biochemicals, Uetersen, Germany) was performed slightly shaking for 30 min at 37 °C. Subsequently, repeated filtration steps (with 100 µm and 40 µm cell strainer; Corning, New York, USA), washing steps with phosphate buffered saline (PBS) containing 10% fetal calf serum (FCS; both PAN Biotech, Aidenbach, Germany) and centrifugation steps followed. Afterwards the final cell pellet was resuspended in cell culture medium (Dulbecco’s Modified Eagle Medium (DMEM) with high glucose and GlutaMAX) containing 1 % penicillin/streptomycin (both: Gibco by Life technologies, Darmstadt, Germany) and 10 % FCS, seeded in cell culture flasks and cultivated at 37 °C in a humidified atmosphere. After 24 h of isolation, the CD34-positive subpopulation was isolated using the Dynal® CD34 precursor cell isolation system (Invitrogen, Karlsruhe, Germany). For this purpose, the non-adherent cells were removed by two washing steps with PBS and incubated with CD34 antibody-coupled magnetic particles in cell culture medium. Non-adherent beads were removed by two washing steps with PBS, followed by trypsination for cell detachment. Repeated steps of magnet exposure and washing with PBS/0.1% FCS on a rotating mixer at 4 °C purified the CD34-positive cell suspension. In passage 4, adMSC were seeded at 20,000 cells/cm^2^ and cultivated over a period of 7 days in DMEM containing 1% penicillin/streptomycin (both: Gibco by Life technologies, Darmstadt, Germany) and 10% FCS (PAN Biotech, Aidenbach, Germany). After 72 h, different concentrations of recombinant human TGF-ß1 (1 ng/mL, 5 ng/mL, and 10 ng/mL; ImmunoTools GmbH, Friesoythe, Germany) in cell culture medium were added to the adMSC cultures. The time of TGF-ß1 addition was defined as day 0. adMSC cultures without TGF-ß1 exposure served as unstimulated controls. If not stated otherwise, all plastic wares were from Greiner Bio-One.

### 4.2. Quantification of the Cell Number and Metabolic Activity 

Quantification of the cell number and metabolic cell activity were performed on days 0, 1, 3, and 7 after the start of the experiment. The cell number was analyzed by crystal violet staining (Sigma-Aldrich, Taufkirchen, Germany) following the instructions previously described [54]. The measurement of the optical density [OD] provides information about the amount of the DNA-bound dye, and thus, relative values for the cell number [55,56]. The determination of the metabolic cell activity was performed using the MTS assay (Promega, Madison, WI, USA). For this purpose, the adMSC were incubated with 3-(4.5-dimethylthiazol-2yl)-5-(3-carboxymethoxyphenyl)-2-(4-sulfophenyl)-2H- tetrazolium for 1 hour in a humidified atmosphere at a temperature of 37°C and 5% CO_2_ content. Here, the NAD (P) H-dependent oxidoreductase reduces the colorless tetrazolium salt into a colored formazan salt, whereby the conversion rate correlates linearly with the number of viable cells. Employing the anthos Mikrosysteme microplate reader (Friesoythe, Germany), the absorbance of the supernatant was measured at 620 nm for the crystal violet staining and at 490 nm with 620 as reference for the MTS assay.

### 4.3. Cell Cycle Analyses

The cell cycle analyses were performed on days 0, 1, 3 and 7 after TGF-ß1 exposure using the Nucleocounter® NC-3000^TM^ (chemometec, Lillerod, Denmark). The two-step cell cycle analysis assay was performed according to the manufacturer’s instructions. For this purpose, the cell culture medium was removed, cells were washed once with 3 mL phosphate buffered saline (PBS, PAN Biotech, Aidenbach, Germany) and incubated with 250 µL solution 10 (lysisbuffer), which contained 10 µg/mL DAPI, for 5 minutes in a humidified atmosphere at a temperature of 37 °C and with a 5 % CO_2_ content. Afterwards, the cells were resuspended thoroughly and supplemented with 250 µL Solution 11 (stabilization buffer). The cellular fluorescence and DNA content were quantified using the NC-Slide A2^TM^ and the NucleoView NC-3000^TM^ software (chemometec, Lillerod, Denmark). The analysis of the cell cycle phases was executed by the Dean-Jett Fox model using FlowJo software Version 10 (Dickinson Company, Becton, NJ, USA). 

### 4.4. Quantification of Mitochondrial Respiration and Glycolysis

The determination of the mitochondrial respiration and glycolysis was performed using the Agilent Seahorse XFp Cell Mito Stress Test (Agilent Technologies, Santa Clara, USA). The test enables a direct measurement of the oxygen consumption rate and the extracellular acidification rate and thus an evaluation of the mitochondrial and glycolytic activity. The modulators of the respiratory chain in this test kit were oligomycin, carbonyl-cyanide-4 (trifluoromethoxy) phenylhydrazone (FCCP) and a complex of rotenone and antimycin A. The first injection step with oligomycin (1.5 µM) inhibited the complex V (ATP synthase) of the respiratory chain, which resulted in a decrease of the oxygen consumption rate (OCR). The second injection with FCCP (0.75 µM), which is an uncoupling agent that disturbs the proton gradient and the mitochondrial membrane potential, led to a maximum OCR by complex IV. The third injection step with a complex (0.5 µM) of rotenone, which is an inhibitor of complex I, and antimycin A, a complex III inhibitor, disrupted the mitochondrial respiration and facilitated the conclusion of non-mitochondrial respiration as result of cellular oxidative reactions not associated with energy metabolism. If not stated otherwise, all reagents and plastic wares were from Agilent Technologies. According to the manufacturer’s instructions, the adMSC were seeded into an 8-well miniplate with a density of 15,000 cells/well (in passage 4). After 72 h of incubation, the adMSC were added with 1 ng/mL and 10 ng/mL TGF-ß1 in cell culture medium, whereas adMSC without the addition of TGF-ß1 served as control cultures. The mitochondrial function was determined 1, 3, and 7 days after TGF-ß1 exposure. All of the following steps were conducted according to the manufacturer’s instructions. The main component of the assay medium was DMEM, containing 1 mM pyruvate, 2 mM glutamine, and 10 mM glucose with a pH-value of 7.4. 

### 4.5. Gene Expression Analyses

#### 4.5.1. RNA Extraction

According to the manufacturer’s instructions, the total RNA extraction from all samples on day one and three were performed using a RNeasy Mini Kit (Qiagen, Hilden, Germany). For this purpose, after 72 h of incubation, the adMSC were added with 10 ng/mL TGF-ß1 in cell culture medium, whereas adMSC without the addition of TGF-ß1 served as control cultures. The RNA quality was checked by the spectrophotometer Nanodrop 1000 (Thermo Fisher Scientifc, Waltham, USA) using the A260/A280 ratio, whereby the RNA integrity was determined by the bioanalyzer Agilent RNA 6000 Nano Kit (Technologies, Agilent, SC, USA). 

#### 4.5.2. Microarray Analysis

Analysis of gene expression was carried out using the Human Clariom S Array (Thermo Fisher Scientifc, Waltham, USA). For the amplification and labeling reactions, 35 µg/mL RNA from the control and TGF-ß1 cultures were appropriated. The hybridization and washing steps of the gene chips were realized according to the manufacturer’s instructions. To read out the microarrays, the GeneChip® 3000 7G laser scanner, controlled by the Affymetrix GeneChip Command Console Software (both Thermo Fisher Schientifc, Waltham, USA) (with a 3 µm resolution, 488 nm excitation and 570 nm emission wavelengths) was used. 

#### 4.5.3. Microarray Data Analysis

Analysis of the microarray data was conducted with the Transcriptome Analysis Console Software (Version 4.0.1) provided by Thermo Fisher [57]. The analysis included quality control, data normalization, and statistical testing for differential expression using the LIMMA method [58]. Transcripts are considered significantly differentially expressed with a fold change (FC) higher than 2 or smaller than -2, a false discovery rate (FDR) of < 0.05 and a *p*-value of < 0.05. The pathway analyses were conducted based on the Wiki-Pathways database. 

#### 4.5.4. Gene Expression Network Analysis

Functional and pathway enrichment analysis of the differentially expressed genes (DEGs) was performed using the EnrichR webtool [27]. Pathways and GO terms with an adjusted *p*-value < 0.05 were considered significantly overrepresented. The KEGG (Kyoto Encyclopedia of Genes and Genomes), BioCarta, Wiki, and Panther pathway databases were used to obtain specific gene annotations [59,60,61]. The results were further visualized using the R package GOplot and GO-Chord [62].

### 4.6. Data Illustration and Statistical Analysis

All analyses include a minimum of three independent cultures of human adMSC with three technical replicates which were compared with their controls. Numerical data in Figure 1, Figure 2b, and Figure 3b are presented as box plots, whereby the boxes indicate interquartile ranges, horizontal lines within the boxes indicate medians, and whiskers indicate minimum and maximum values. Data in Figure 2a and Figure 3a are presented as means with the standard error of the mean (SEM). Since the data received were distributed normally (Shapiro-Wilk test), the statistical significance within the dataset was calculated with the Two-Way ANOVA followed by Dunnett’s multiple comparison post hoc using GraphPad Prism Version 7.00 for Windows (GraphPad Software, San Diego, USA) with a *p*-value of 0.05. The statistical significance between datasets for data which was not normally distributed was calculated with the One-Way ANOVA by Dunnett’s multiple comparison post hoc or Two-Way ANOVA by Tukey’s multiple comparison post hoc, both with a *p*-value of 0.05. 

### 4.7. Ethic Statement

The local ethical committee (Rostock University Medical Center) approved this study under the registration number A2019-0107. All experiments were conducted after receiving full consent of the patients.

## Figures and Tables

**Table 1:** Cell cycle analysis after the addition of 10 ng TGF-ß1/ml compared with the control cultures. Data depicted as mean with the standard error of the mean (SEM) as percentage of all cells. Since the dataset did not represent a Gaussian distribution (Shapiro-Wilk test), the statistical analysis was performed using the Two-Way ANOVA test followed by Dunnett’s multiple comparison post hoc test (*n* = 4). * *p* ≤ 0.05.

		*Cell Cycles Phases*			
		sub G1	G0/G1	S	G2
*0 d*	[%]	1.4 (± 0.1)	89.5 (± 1.3)	2.3 (± 0.6)	6.1 (± 0.5)
*1 d*	control [%]	1.9 (± 0.4)	88.7 (± 1.3)	2.9 (± 0.3)	6.0 (± 0.9)
	TGF-ß1 [%]	0.9 (± 0.5)	86.1 (± 3.9)	5.7 (± 3.1)	4.1 (± 1.6)
*3 d*	control [%]	2.3 (± 0.2)	89.3 ( ±1.0)	2.5 (± 0.5)	5.8 (± 0.7)
	TGF-ß1 [%]	0.9 (± 0.3)	89.4 (± 1.2)	2.0 (± 0.4)	6.3 (± 0.9)
*7 d*	control [%]	2.5 (± 0.2)	89.9 (± 0.8)	2.3 (± 0.5)	5.5 (± 0.5)
	TGF-ß1 [%]	1.5 (± 0.2)	87.9 (± 1.7)	3.4 (± 1.0)	6.2 (± 0.5)

**Table 2:** Energy metabolism and amino acid metabolism with the significantly differentially expressed genes.

Energy Metabolism		Amino Acid Metabolism	
Upregulated	Downregulated	Upregulated	Downregulated
PPP3CA	PPARG	ACLY	ADH1C
PPP3R1	MEF2C	ALDH18A1	ALDH1A1
PPP3CC	NRF1	AOC3	ALDH7A1
PPARD	PPARGC1A	ARG2	AUH
PRKAB2	SIRT3	ASNS	FAH
PRKAG2	TFAM	BCAT1	GLUL
		CBS	HIBADH
		CTH	MCCC1
		EPRS	PDK4
		GCLM	PPM1L
		GLS	
		GOT1	
		GPT2	
		GSR	
		IARS	
		ODC1	
		P4HA2	
		PYCR1	
		SRM	
		WARS	

Abbreviations: PPP3CA: protein phosphatase 3 catalytic subunit alpha; PPP3R1; protein phosphatase 3 regulatory subunit beta; PPP3CC: protein phosphatase 3 catalytic subunit gamma; PPARD: peroxisome proliferator-activated receptor delta; PRKAB2: protein kinase AMP-activated non-catalytic subunit beta 2; PRKAG2: proteinkinase AMP-activated non-catalytic subunit gamma 2; PPARG: peroxisome proliferator-activated receptor gamma; MEF2C: myocyte enhancer factor 2 C; NRF1: nuclear respiratory factor 1; PPARGC1A: peroxisome proliferator-activated receptor gamma Coactivator 1 alpha; SIRT3: Sirtuin 3; TFAM: transcription factor A, mitochondrial; ACLY: ATP-citrate lyase; ALDH18A1: aldehyde dehydrogenase 18 family member A1; AOC3: amine oxidase copper containing 3; ARG2: arginase 2; ASNS: asparagine synthetase; BCAT1: branched chain amino-acid transaminase 1; CBS: cystathionine beta-synthetase; CTH: cystathionine gamma-lyase; EPRS: glutamyl-prolyl-tRNA synthetase; GCLM: glutamate-cysteine ligase; GLS: glutaminase; GOT1:glutamic-oxaloacetic transaminase 1; GPT2: glutamic pyruvate transaminase; GSR: glutathionine reductase; IARS: isoleucyl-tRNA synthetase; ODC1: ornithine decarboxylase; P4HA2: prolyl 4-hydroxylase subunit alpha 2; PYCR1: pyrroline-5-carboxylate reductase1; SRM: spermidine synthetase; WARS: tryptophanyl-tRNA synthetase; ADH1C: alcohol dehydrogenase 1C, gamma polypeptide; ALDH1A1: aldehyde dehydrogenase 7 family member A1; ALDH7A1: aldehyde dehydrogenase 1 family member A1; AUH: AU RNA binding methylglutaconyl CoA hydratase; FAH: fumarylacetoacetate hydrolase; GLUL: glutamate-ammonia ligase; HIBADH: 3-hydroxyisobutyrate dehydrogenase; MCCC1: methylcrotonoyl-CoA carboxylase 1; PDK4: pyruvate dehydrogenase kinase 4; PPM1L: protein phosphatase, Mg^2+^/Mn^2+^ dependent 1L.

**Figure A1:**
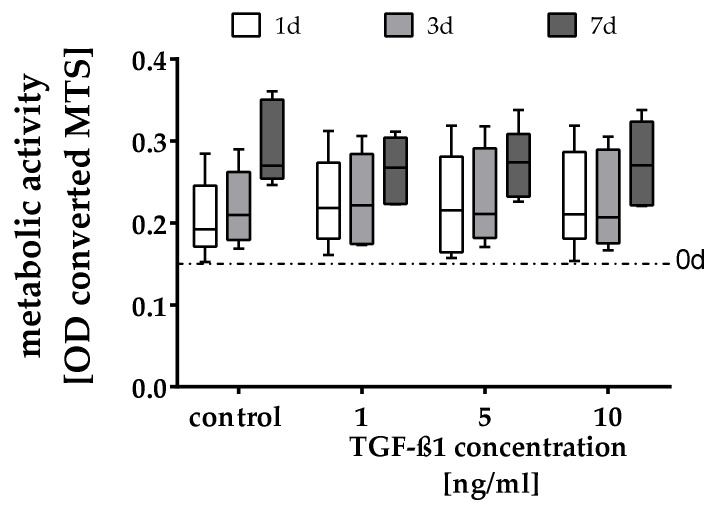
Optical density of metabolic activity after exposure to different concentrations of TGF-ß1 on day 0, 1, 3, and 7 (metabolic activity measured via MTS conversion assay). Data presented as box plots with medians, interquartile ranges, and minimum/maximum values (*n* = 6). Since the dataset did not represent a Gaussian distribution (Shapiro-Wilk test), the statistical analysis was performed using the Two-Way ANOVA test followed by Dunnett’s multiple comparison post hoc test. * *p* ≤ 0.05. Comparison with the control.

**Figure A2:**
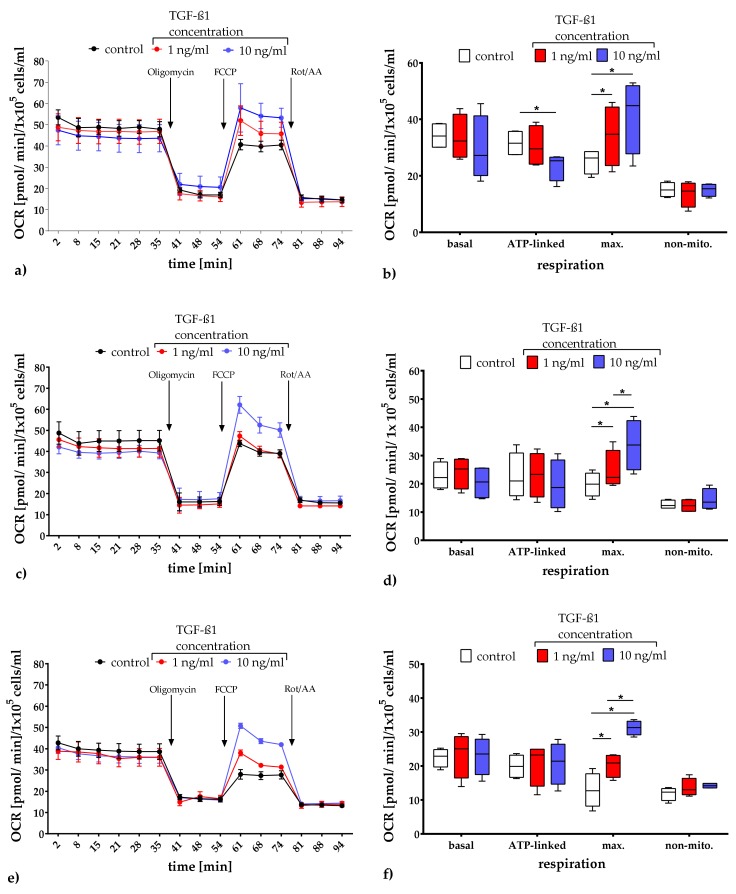
Quantification of the oxygen consumption rate (OCR) after TGF-ß1 exposure on days 1 (a, b), 3 (c, d), and 7 (e, f). OCR data presented as mean with the standard error of the mean normalized to 1 x 10^5^ cells/ml (a, c, e). Corresponding to their function in the respiratory chain, the numerical data were presented as box plots, with medians, interquartile ranges, and minimum/maximum values (b, d, f). Since the dataset did not represent a Gaussian distribution (Shapiro-Wilk test), the statistical analysis was performed using the Two-Way ANOVA test followed by Tukey’s multiple comparison post hoc test (*n* = 4). *, *p*-value ≤ 0.05. Comparison with the control. FCCP: carbonyl-cyanide-4 (trifluoromethoxy) phenylhydrazone; Rot/AA: rotenone/antimycin A; ATP: adenosine triphosphate; max.: maximal; non-mito.: non-mitochondrial.

**Figure A3:**
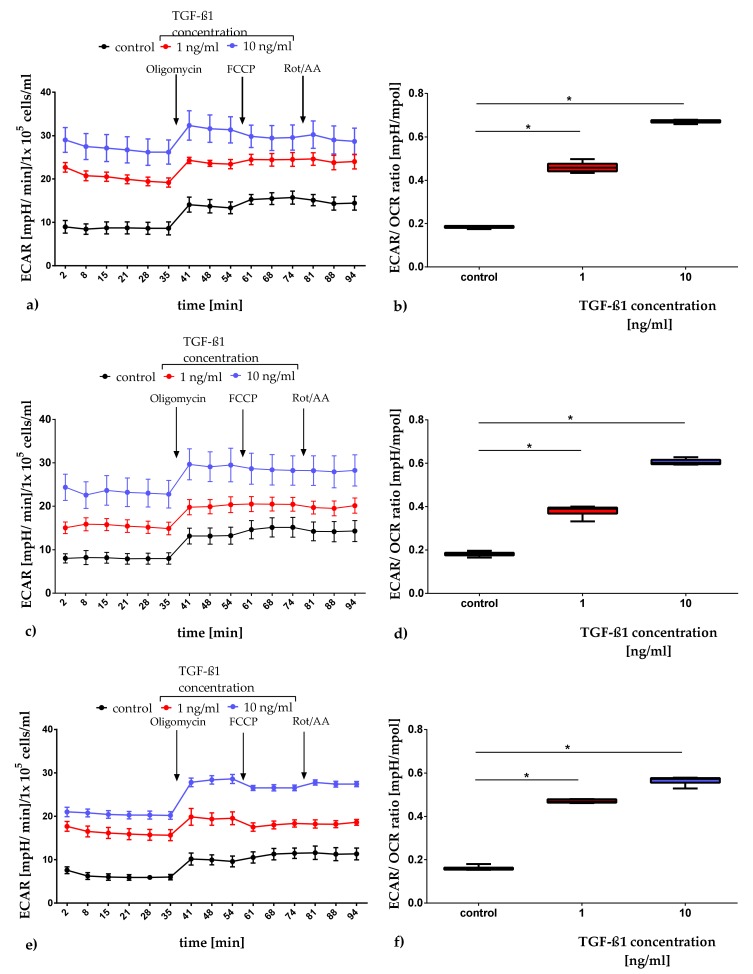
Quantification of the extracellular acidification rate (ECAR) after TGF-ß1 exposure on day 1 (a, b), 3 (c, d), and 7 (e, f). ECAR data are presented as a mean, with the standard error of the mean normalized to 1 x 10^5^ cells/ml (a, c, e). The basal metabolism of the cultures was assessed by calculating ECAR/OCR ratios. Data were presented as box plots, with medians, interquartile ranges, and minimum/maximum values (b, d, f). Since the dataset did not represent a Gaussian distribution (Shapiro-Wilk test), the statistical analysis was performed using the One-Way ANOVA test followed by Dunnett’s multiple comparison post hoc test (n= 4). *, *p*-value ≤ 0.05. Comparison to the control. FCCP: carbonyl-cynaide-4 (trifluoromethoxy) phenylhydrazone; Rot/AA: rotenone/antimycin A; ATP: adenosine triphosphate; max.: maximal.

## References

[1] Klaus S. (2004). Adipose tissue as a regulator of energy balance. Curr. Drug Targets.

[2] Rosen E.D., Spiegelman B.M. (2006). Adipocytes as regulators of energy balance and glucose homeostasis. Nature.

[3] Barchetta I., Cimini F.A., Ciccarelli G., Baroni M.G., Cavallo M.G. (2019). Sick fat: The good and the bad of old and new circulating markers of adipose tissue inflammation. J. Endocrinol. Investig..

[4] Bourin P., Bunnell B.A., Casteilla L., Dominici M., Katz A.J., March K.L., Redl H., Rubin J.P., Yoshimura K., Gimble J.M. (2013). Stromal cells from the adipose tissue-derived stromal vascular fraction and culture expanded adipose tissue-derived stromal/stem cells: A joint statement of the International Federation for Adipose Therapeutics and Science (IFATS) and the International Society for Cellular Therapy (ISCT). Cytotherapy.

[5] Tallone T., Realini C., Böhmler A., Kornfeld C., Vassalli G., Moccetti T., Bardelli S., Soldati G. (2011). Adult human adipose tissue contains several types of multipotent cells. J. Cardiovasc. Transl. Res..

[6] Zimmerlin L., Donnenberg V.S., Pfeifer M.E., Meyer E.M., Péault B., Rubin J.P., Donnenberg A.D. (2010). Stromal vascular progenitors in adult human adipose tissue. Cytom. Part A J. Int. Soc. Anal. Cytol..

[7] Zuk P.A., Zhu M., Mizuno H., Huang J., Futrell W., Katz A.J., Benhaim P., Lorenz P., Hedrick M.H. (2001). Multilineage cells from human adipose tissue: Implications for cell-based therapies. Tissue Eng..

[8] Grafe I., Alexander S., Peterson J.R., Snider T.N., Levi B., Lee B., Mishina Y. (2019). TGF-β family signaling in mesenchymal differentiation. Cold Spring Harb. Perspect. Biol..

[9] Lin G., Garcia M., Ning H., Banie L., Guo Y.-L., Lue T.F., Lin C.-S. (2008). Defining stem and progenitor cells within adipose tissue. Stem Cells Dev..

[10] Montserrat E.R. (2014). Adipose tissue: Cell heterogeneity and functional diversity. Endocrinol. Nutr. (Engl. Ed.).

[11] Löffler G., Hauner H. (1987). Adipose tissue development: The role of precursor cells and adipogenic factors. Klin. Wochenschr..

[12] Assian R.K., Komoriya A., Meyers C.A., Miller D.M., Sporn M.B. (1983). Transforming growth factor-ß in human platelets: Identifcation of a major storage site, purification and characterization. J. Biol. Chem..

[13] Ibelgaufts H. (1992). Lexikon Zytokine: Mit Tabellen.

[14] De Farias A.V., Carrillo-Gálvez A.B., Martín F., Anderson P. (2018). TGF-β and mesenchymal stromal cells in regenerative medicine, autoimmunity and cancer. Cytokine Growth Factor Rev..

[15] Ng M., Fareha A., Ooi S.I., Aminuddin B.S., Ruszymah B.H.I. (2012). Effects of TGF-ß1 on bone marrow stem cells proliferation, osteogenic differentiation and maturation. Regen. Res..

[16] Walenda G., Abnaof K., Joussen S., Meurer S., Smeets H., Rath B., Hoffmann K., Fröhlich H., Zenke M., Weiskirchen R. (2013). TGF-beta1 does not induce senescence of multipotent mesenchymal stromal cells and has similar effects in early and late passages. PLoS ONE.

[17] Zhou W., Park I., Pins M., Kozlowski J.M., Jovanovic B., Zhang J., Lee C., Ilio K. (2003). Dual regulation of proliferation and growth arrest in prostatic stromal cells by transforming growth factor-ß1. Endocrinology.

[18] Hinz B., Phan S.H., Thannickal V.J., Galli A., Bochaton-Piallat M., Gabbiani G. (2007). The myofibroblast: One function, multiple origins. Am. J. Pathol..

[19] Hinz B., Celetta G., Tomasek J.J., Gabbiani G., Chaponnier C. (2001). Alpha-smooth muscle actin expression upregulates fibroblast contractile activity. Mol. Biol. Cell.

[20] Hinz B., Gabbiani G., Chaponnier C. (2002). The NH2-terminal peptide of alpha-smooth muscle actin inhibits force generation by the myofibroblast in vitro and in vivo. J. Cell Biol..

[21] Eyden B. (2008). The myofibroblast: Phenotypic characterization as a prerequisite to understanding its functions in translational medicine. J. Cell. Mol. Med..

[22] Tomasek J.J., Gabbiani G., Hinz B., Chaponnier C., Brown R.A. (2002). Myofibroblasts and mechano-regulation of connective tissue remodelling. Nat. Rev. Mol. Cell Biol..

[23] Ochocki J.D., Simon M.C. (2013). Nutrient-sensing pathways and metabolic regulation in stem cells. J. Cell Biol..

[24] Shyh-Chang N., Daley G.Q. (2015). Metabolic switches linked to pluripotency and embryonic stem cell differentiation. Cell Metab..

[25] Meyer J., Salamon A., Mispagel S., Kamp G., Peters K. (2018). Energy metabolic capacities of human adipose-derived mesenchymal stromal cells in vitro and their adaptations in osteogenic and adipogenic differentiation. Exp. Cell Res..

[26] Shares B.H., Busch M., White N., Shum L., Eliseev R.A. (2018). Active mitochondria support osteogenic differentiation by stimulating β-catenin acetylation. J. Biol. Chem..

[27] Chen E.Y., Tan C.M., Kou Y., Duan Q., Wang Z., Meirelles G.V., Clark N.R., Ma’ayan A. (2013). Enrichr: Interactive and collaborative HTML5 gene list enrichment analysis tool. BMC Bioinform..

[28] Jian H., Shen X., Liu I., Semenov M., He X., Wang X. (2006). Smad3-dependent nuclear translocation of ß-catenin is required for TGF-ß1- induced proliferation of bone marrow-derived adult human mesenchymal stem cells. Genes Dev..

[29] Koli K., Ryynänen M.J., Keski-Oja J. (2008). Latent TGF-beta binding proteins (LTBPs)-1 and -3 coordinate proliferation and osteogenic differentiation of human mesenchymal stem cells. Bone.

[30] Ng F., Boucher S., Koh S., Sastry K.S.R., Chase L., Lakshmipathy U., Choong C., Yang Z., Vemuri M.C., Rao M.S. (2008). PDGF, TGF-beta, and FGF signaling is important for differentiation and growth of mesenchymal stem cells (MSCs): Transcriptional profiling can identify markers and signaling pathways important in differentiation of MSCs into adipogenic, chondrogenic, and osteogenic lineages. Blood.

[31] Debacq-Chainiaux F., Borlon C., Pascal T., Royer V., Eliaers F., Ninane N., Carrard G., Friguet B., de Longueville F., Boffe S. (2005). Repeated exposure of human skin fibroblasts to UVB at subcytotoxic level triggers premature senescence through the TGF-beta1 signaling pathway. J. Cell Sci..

[32] Ito T., Sawada R., Fuijiwara Y., Seyama Y., Tsuchiya T. (2007). FGF-2 suppresses cellular senescence of human mesenchymal stem cells by down-regulation of TGF-β2. Biochem. Biophys. Res. Commun..

[33] Massagué J. (2000). How cells read TGF-ß signals. Nat. Rev. Mol. Cell Biol..

[34] Kassem M., Keivborg M., Eriksen E.F. (2000). Production and action of transforming growth factor-ß in human osteoblast cultures: Dependence on cell differentiation and modulation by calcitriol. Eur. J. Clin. Investig..

[35] Roostaeian J., Carlsen B., Simhaee D., Jarrahy R., Huang W., Ishida K., Rudkin G.H., Yamaguchi D.T., Miller T.A. (2006). Characterization of growth and osteogenic differentiation of rabbit bone marrow stromal cells. J. Surg. Res..

[36] Morton J.C., Armstrong J.A., Sud A., Tepikin A.V., Sutton R., Criddle D.N. (2019). Altered bioenergetics of blood cell sub-populations in acute pancreatitis patients. J. Clin. Med..

[37] Valente A.J., Fonseca J., Moradi F., Foran G., Necakov A., Stuart J.A. (2019). Mitochondria in health and in sickness: Quantification of mitochondrial network characteristics in health and disease. Mitochondria in Health and in Sickness.

[38] Vander Heiden M.G., Cantley L.C., Thompson C.B. (2009). Understanding the warburg effect: The metabolic requirements of cell proliferation. Science.

[39] Peters K., Kamp G., Berz A., Unger R.E., Barth S., Salamon A., Rychyly J., Kirckpatrick C.J. (2009). Changes in human endothelial cell energy metabolic capacities during in vitro cultivation. The role of “aerobic glycolysis” and proliferation. Cell. Physiol. Biochem..

[40] Meyer J., Engelmann R., Kamp G., Peters K. (2019). Human adipocytes and CD34+ cells from the stromal vascular fraction of the same adipose tissue differ in their energy metabolic enzyme configuration. Exp. Cell Res..

[41] Tian W.N., Braunstein L.D., Pang J., Stuhlmeier K.M., Xi Q.C., Tian X., Stanton R.C. (1998). Importance of glucose-6-phosphate dehydrogenase activity for cell growth. J. Biol. Chem..

[42] Warbug O. (1956). On the origin of cancer cells. Science.

[43] DeBerardinis R.J., Lum J.J., Hatzivassiliou G., Thompson C.B. (2008). The biology of cancer: Metabolic reprogramming fuels cell growth and proliferation. Cell Metab..

[44] Mischen B.T., Follmar K.E., Moyer K.E., Buehrer B., Olbrich K.C., Levin L.S., Klitzman B., Erdmann D. (2008). Metabolic and functional characterization of human adipose-derived stem cells in tissue engineering. Plast. Reconstr. Surg..

[45] Lunt S.Y., Vander Heiden M.G. (2001). Aerobic glycolysis: Meeting the metabolic requirements of cell proliferation. Annu. Rev. Cell Dev. Biol..

[46] Landrier J.-F., Kasiri E., Karkeni E., Mihály J., Béke G., Weiss K., Lucas R., Aydemir G., Salles J., Walrand S. (2017). Reduced adiponectin expression after high-fat diet is associated with selective up-regulation of ALDH1A1 and further retinoic acid receptor signaling in adipose tissue. Fed. Am. Soc. Exp. Biol..

[47] Haenisch M., Treuting P.M., Brabb T., Goldstein A.S., Berkseth K., Amory J.K., Paik J. (2018). Pharmacological inhibition of ALDH1A enzymes suppresses weight gain in a mouse model of diet-induced obesity. Obes. Res. Clin. Pract..

[48] Lu G., Ota A., Ren S., Franklin S., Rau C.D., Ping P., Lane T.F., Zhou Z.H., Reue K., Lusis A.J. (2008). PPM1l encodes an inositol requiring-protein 1 (IRE1) specific phosphatase that regulates the functional outcome of the ER stress response. Mol. Metab..

[49] Chen Y., Zhu J., Lum P.Y., Yang X., Pinto S., MacNeil D.J., Zhang C., Lamb J., Edwards S., Sieberts S.K. (2008). Variations in DNA elucidate molecular networks that cause disease. Nature.

[50] Hao Q., Yadav R., Basse A.L., Petersen S., Sonne S.B., Rasmussen S., Zhu Q., Lu Z., Wang J., Audouze K. (2014). Transcriptome profiling of brown adipose tissue during cold exposure reveals extensive regulation of glucose metabolism. Am. J. Physiol. Endocrinol. Metab..

[51] Buresova J., Janovska P., Kuda O., Krizova J., der Stelt I.R., Keijer J., Hansikova H., Rossmeisl M., Kopecky J. (2018). Postnatal induction of muscle fatty acid oxidation in mice differing in propensity to obesity: A role of pyruvate dehydrogenase. Int. J. Obes..

[52] Kumar D., Bansal G., Narang A., Basak T., Abbas T., Dash D. (2016). Integrating transcriptome and proteome profiling: Strategies and applications. Proteomics.

[53] Peters K., Salamon A., van Vlierberghe S., Rychly J., Kreutzer M., Neumann H.G., Schacht E., Dubruel P. (2009). A new approach for adipose tissue regeneration based on human mesenchymal stem cells in contact to hydrogels-an in vitro study. Adv. Eng. Mater..

[54] Salamon A., Jonitz-Heincke A., Adam S., Rychly J., Müller-Hilke B., Bader R., Lochner K., Peters K. (2013). Articular cartilage-derived cells hold a strong osteogenic differentiation potential in comparison to mesenchymal stem cells in vitro. Exp. Cell Res..

[55] Gillies R., Didier N., Denten M. (1986). Determination of cell number in monolayer cultures. Anal. Biochem..

[56] Noeske K. (1966). Die Bindung von Kristallviolett an Desoxyribonukleinsäure. Histochemie.

[57] Thermo Fisher Scientific (2019). Transcriptome Analysis Console (TAC) Software.

[58] Ritchie M.E., Phipson B., Di W., Hu Y., Law C.W., Shi W., Smyth G.K. (2015). limma powers differential expression analyses for RNA-sequencing and microarray studies. Nucleic Acids Res..

[59] Ogata H., Goto S., Sato K., Fujibuchi W., Bono H., Kanehisa M. (1999). KEGG: Kyoto Encyclopedia of Genes and Genomes. Nucleic Acids Res..

[60] Pico A.R., Kelder T., van Iersel M.P., Hanspers K., Conklin B.R., Evelo C. (2008). WikiPathways: Pathway editing for the people. PLoS Biol..

[61] Thomas P.D., Campbell M.J., Kejariwal A., Mi H., Karlak B., Daverman R., Diemer K., Muruganujan A., Narechania A. (2003). PANTHER: A library of protein families and subfamilies indexed by function. Genome Res..

[62] Walter W., Sánchez-Cabo F., Ricote M. (2015). GOplot: An R package for visually combining expression data with functional analysis. Bioinformatics.

